# Inflammatory and myeloid-associated gene expression before and one day after infant vaccination with MVA85A correlates with induction of a T cell response

**DOI:** 10.1186/1471-2334-14-314

**Published:** 2014-06-09

**Authors:** Magali Matsumiya, Stephanie A Harris, Iman Satti, Lisa Stockdale, Rachel Tanner, Matthew K O’Shea, Michelle Tameris, Hassan Mahomed, Mark Hatherill, Thomas J Scriba, Willem A Hanekom, Helen McShane, Helen A Fletcher

**Affiliations:** 1 Jenner Institute, University of Oxford, Old Road Campus Research Building, Oxford, UK; 2 South African Tuberculosis Vaccine Initiative, Institute of Infectious Disease and Molecular Medicine and School of Child and Adolescent Health, University of Cape Town, Cape Town, South Africa; 3 Division of Community Health, Stellenbosch University, Stellenbosch, South Africa; 4 Metropolitan District Health Services, Western Cape, Government Health, Cape Town, South Africa; 5 Current affiliation: London School of Hygiene and Tropical Medicine, Keppel St, London WC1E 7HT, UK

**Keywords:** Tuberculosis, Vaccine, Innate immunity, Transcriptomics, MVA85A

## Abstract

**Background:**

Tuberculosis (TB) remains a global health problem, with vaccination likely to be a necessary part of a successful control strategy. Results of the first Phase 2b efficacy trial of a candidate vaccine, MVA85A, evaluated in BCG-vaccinated infants were published last year. Although no improvement in efficacy above BCG alone was seen, cryopreserved samples from this trial provide an opportunity to study the immune response to vaccination in this population.

**Methods:**

We investigated blood samples taken before vaccination (baseline) and one and 28 days post-vaccination with MVA85A or placebo (Candin). The IFN-γ ELISpot assay was performed at baseline and on day 28 to quantify the adaptive response to Ag85A peptides. Gene expression analysis was performed at all three timepoints to identify early gene signatures predictive of the magnitude of the subsequent adaptive T cell response using the significance analysis of microarrays (SAM) statistical package and gene set enrichment analysis.

**Results:**

One day post-MVA85A, there is an induction of inflammatory pathways compared to placebo samples. Modules associated with myeloid cells and inflammation pre- and one day post-MVA85A correlate with a higher IFN-γ ELISpot response post-vaccination. By contrast, previous work done in UK adults shows early inflammation in this population is not associated with a strong T cell response but that induction of regulatory pathways inversely correlates with the magnitude of the T cell response. This may be indicative of important mechanistic differences in how T cell responses develop in these two populations following vaccination with MVA85A.

**Conclusion:**

The results suggest the capacity of MVA85A to induce a strong innate response is key to the initiation of an adaptive immune response in South African infants but induction of regulatory pathways may be more important in UK adults. Understanding differences in immune response to vaccination between populations is likely to be an important aspect of developing successful vaccines and vaccination strategies.

**Trial registration:**

ClinicalTrials.gov number NCT00953927

## Background

Tuberculosis (TB) is a major global health problem with an estimated 8.6 million new cases and 1.3 million deaths in 2012 [1]. Effective vaccination is likely to be necessary for the long-term control of the TB epidemic however Bacille Calmette-Guerin (BCG), the only currently licensed vaccine, provides variable protection against pulmonary disease [2]. Despite high BCG coverage, the incidence of TB remains high in endemic countries. Research efforts into new TB vaccines have focused largely on two strategies; either to modify BCG or replace it with an attenuated strain of *Mycobacterium tuberculosis* (*M.tb),* or to improve the protection provided by BCG through prime-boost regimes, often using viral vectors expressing TB antigens to enhance the pool of circulating memory cells primed by vaccination with BCG [3]. Twelve novel TB vaccines are currently in clinical trials, including two in Phase 2b efficacy studies [1]. The results of the first efficacy trial of a novel vaccine, Modified Vaccinia virus Ankara expressing antigen 85A (MVA85A) were published in early 2013 [4].

Although the efficacy of boosting BCG with MVA85A was not superior to that of BCG alone, the study demonstrated that a trial of a novel TB vaccine is feasible in a high burden setting. Furthermore, the collection of blood samples from all infants during the trial will enable research into the mechanisms of disease risk and response to vaccination in this setting. Previous studies with MVA85A have shown it to be safe and immunogenic in several diverse populations including adults without or with latent TB infection in the UK; healthy, latently infected and HIV-infected adults in Africa and healthy adolescents, children and infants in Africa [5-8]. The vaccine shows a quantitatively lower immunogenicity in African adults and in younger children and infants in Africa compared to UK adults [4]. In all trials to date, MVA85A induces antigen-specific Th1 and Th17 cells, believed to be important in protection against tuberculosis [9-11]. If, as has been suggested [12], low T cell responses to vaccination in this trial contributed to the lack of vaccine efficacy, understanding the mechanisms determining the magnitude of the response to vaccination is important to the development of an improved vaccine.

Several studies published in the last five years have demonstrated the power of genomics approaches in understanding the molecular mechanisms of the immune response to vaccination [13-16]. Work using the yellow fever vaccine, YF-17D, identified a gene expression signature in circulating leukocytes of vaccinated volunteers shortly after vaccination which could predict the magnitude of the subsequent CD8+ T cell response [13]. Follow-up studies have yielded further mechanistic insight, showing that activation of the nutrient sensor GCN2 in dendritic cells following vaccination leads to increased antigen presentation and the development of a stronger immune response [17]. Similar approaches have been used by other groups and are beginning to reveal some of the factors contributing to the variability of the human immune response. The data show the importance of innate pathways in determining the magnitude of subsequent adaptive immune responses with a role for the stress response and gut microbiota in particular [18]. The cellular environment and its modification by vaccines and adjuvants are determined by many factors, underscoring the large variation seen in the immune response to vaccines across individuals and populations.

Understanding the differences in immune responses in different groups is key to developing targeted approaches to vaccination. The immune system changes with age, with a decrease in response to vaccination often noted in the elderly [19], though a recent study found no decrease in T cell responses in older adults following a novel MVA-vectored influenza vaccine [20]. Inflammation, apoptosis and immune senescence have all been linked to lower responses to vaccination in this age group [16,21]. As the population of the developed world continues to age, understanding these factors will be important in developing effective vaccination strategies. At the other end of the spectrum are the immature immune systems of infants, which also differ to those of adults [8,22]. Although children and infants, particularly in the developing world, are the target population for many vaccines against infectious diseases, the factors underpinning the immune response to vaccination in this population remain poorly characterized. Understanding the immune response to vaccination in infants living in areas with a high burden of disease, and how this differs from the immune response of healthy, young adults living in areas of lower disease prevalence –but in whom early testing of vaccines is usually carried out- is therefore a crucial component in the development and early selection of many of the vaccines in development.

In this study, we have analysed gene expression signatures pre- and post-vaccination in infants from the MVA85A Phase 2b efficacy trial who did not develop TB disease during the trial (non-cases) and correlated these changes to the antigen-specific T cell response to vaccination, as measured by IFN-γ ELISpot to Ag85A peptides, in an effort to understand the variability in response to vaccination in this setting. Finally, we compare these findings with previous work performed in UK adults receiving the same vaccine [23], in an effort to characterise some of the differences between these populations.

## Methods

### Origin of samples

Samples used in these experiments were cryopreserved peripheral blood mononuclear cells (PBMC) or whole blood in RNA lysis buffer from a double-blind, randomised, placebo-controlled Phase 2b efficacy trial of a candidate TB vaccine, MVA85A, in BCG-vaccinated, HIV-negative South African infants (South African National Clinical Trials Register DOH-27-0109-2654, ClinicalTrials.gov NCT00953927). Infants were randomized to receive either one dose of MVA85A (1 × 10^8^ plaque forming units in 0.06 mL) or an equal volume of Candida skin test antigen (Candin, AllerMed, USA) as placebo at 4–6 months of age [4]. The trial was approved by the University of Cape Town Faculty of Health Sciences Human Research Ethics Committee, Oxford University Tropical Research Ethics Committee, and the Medicines Control Council of South Africa. Parents or legal guardians provided written, informed consent. Storage of samples for exploratory immunological analyses was fully ethically approved.

The samples used in this study were selected from a subset of 100 infants who had a small blood sample taken one day post-vaccination. The samples were selected to exclude cases (infants who went on to develop TB disease) and controls (selected to demographically match the cases), which will be used in a future study looking at correlates of risk of TB disease. Therefore none of the samples used in this study were taken from infants diagnosed with TB during the course of the trial. Cells were collected 0–7 days pre- and 28 days post-vaccination with MVA85A/placebo in cell preparation tubes with sodium heparin (CPT; Vacutainer; BD) and PBMC separated and cryopreserved. The cells were thawed and stimulated as detailed below. One day post-vaccination, 50-300 μL of whole blood was collected directly via heel prick into a tube filled with RLT buffer (RNeasy kit, Qiagen) containing 10 μL/mL β-mercaptoethanol using a BD Quikheel Lancet. The sample was immediately frozen and RNA extracted as detailed below.

### Cell thawing

Cryopreserved PBMC were rapidly thawed in a 37°C waterbath and transferred to a 15 mL Falcon tube containing 10 mL R10 (RPMI, 10% FCS, 1% L-glutamine, 1% Pen-Strep and 1% sodium pyruvate). PBMC were pelleted, supernatants discarded and resuspended in 10 mL R10 with 20 μL Benzonase (Merck Chemicals Ltd.) and rested overnight at 37°C, 5% CO_2_. PBMC were counted on a Casy Counter (Roche) and split into appropriate volumes for each assay. Not all assays were performed on all samples.

### *Ex-vivo* IFN-γ ELISpot assay

The ex-*vivo* IFN-γ ELISpot assay was performed on thawed PBMC samples collected pre-vaccination and 28 days post-vaccination as previously described [23]. 3 × 10^5^ PBMC were stimulated in triplicate with a pool of Ag85A peptides, consisting of 66 15mers, overlapping by 10 amino acids (2 μg/ml) (Peptide Protein Research).

### Gene expression assays

PBMC from pre-vaccination and 28 days post-vaccination were incubated for 12 hours with either R10 media alone (unstimulated) or pooled Ag85A peptides as described for the ELISpot (2 μg/mL). After 12 hours supernatant was removed and the PBMC resuspended in 350uL RLT buffer (Qiagen) containing 10 μL/mL β-mercaptoethanol and frozen at -20C.

Blood in RLT buffer was thawed and RNA extracted using the RNeasy kit (Qiagen) according to manufacturer’s instructions, including the optional protocol for DNA digest (RNase-free DNase kit, Qiagen). The protocol was modified in the following way for the heelprick samples due to the small volume of whole blood collected: 80% ethanol was added to precipitate the RNA (rather than the recommended 70%) and an extra wash with 350 μL RW1 buffer was performed prior to DNA digest.

Messenger RNA was amplified from the total RNA using the Illumina Totalprep kit (Ambion) according to manufacturer’s instructions. RNA quantity and quality was assessed using a Nanodrop ND-1000 Spectrophotometer and an Agilent Bioanalyser (Agilent RNA 6000 Nano Kit).

750 ng amplified cRNA was labeled and hybridized to Illumina Human HT-12 v4 beadchips as specified in the manufacturer’s instructions. Beadchips were scanned on an Illumina iScan machine and data extracted using the GenomeStudio software.

RNA-Seq was performed on pre- and post-vaccination PBMC from 6 infants (12 samples). Total RNA was sent to the Beijing Genomics Institute (BGI). Libraries were constructed using the TruSeq kit (Illumina) and sequenced on a HiSeq2000 sequencer, using paired-end reads of 90 bp and 30 M sequencing depth. Quality control was performed and the reads aligned to the genome hg19, downloaded from UCSC ( http://genome.ucsc.edu/), using SOAPaligner2.21 with the following constraints: maximum number of mismatches allowed on one read is 5 bp, no gaps allowed, only repeat hits reported.

### Data analysis

#### Ex vivo IFN-γ ELISpot assay

Phytohaemagglutinin (PHA) (Sigma) was used as a positive control and unstimulated wells were used as a measure of background IFN-γ production. Results are reported as spot forming cells (SFC) per million PBMC, calculated by subtracting the mean of the unstimulated wells from the mean of triplicate antigen wells and correcting for the number of PBMC in the well. An ELISpot response was deemed positive if the average count in the positive control wells was at least twice that in the negative control wells and at least 5 spots more than the negative control wells.

#### Illumina microarray

The R package arrayQualityMetrics [24] was used to assess sample quality. 82 heelprick samples and all PBMC samples passed quality control. The R package limma was used to perform background correction and normalization and the gene list was filtered using the gene filter package to remove genes with an expression IQR < 0.3 (log2 transformed). Lists of differentially expressed genes were generated using limma (p-value cut-off of 0.05 after Benjamini-Hochberg correction [25-27]).

The package Significance Analysis of Microarrays (SAM) was used to rank genes correlating with the IFN-γ ELISpot response according to the strength of the correlation [28-30]. The ranked gene list was then inputted into Broad Institute gene set enrichment analysis programme (GSEA) [31,32] as an externally supplied preranked list. The reference gene set used was the Blood Transcription Modules compiled by Li et al. [33]. The significance of module enrichment was assessed by permutation in the GSEA program.

All heatmaps were generated in R, using Euclidean distance and average linkage as methods to calculate the distance matrix and hierarchical clustering respectively. Where correlations are shown, these use the Pearson product–moment correlation coefficient.

#### RNA sequencing

Genes for which the reads per kilobase per million value (rpkm) was <1 in over 40% of samples were excluded. RPKM was calculated as (10^9*C)/(N*L) where C = number of reads uniquely mapped to transcript, N = total number of uniquely mapped reads in sample and L = maximum length of transcript. Raw counts were then analysed in the R package limma and converted to log2 transformed counts per million. These values were compared to the gene expression values for the equivalent samples obtained by microarray analysis.

### Accession codes

Gene expression omnibus: GSE56559 (day 1 heelprick, South African infants), GSE56561 (PBMC, South African infants) GSE40719 (UK adults).

## Results and discussion

Table 1 shows the number of samples used in each assay in this study.

**Table 1:** Samples processed for each assay as part of this study

**Assay**	**Samples processed**	**No. of infants**
GEX: unstimulated PBMC days 0 and 28	60	30
GEX: whole blood day 1	82	82
GEX: Ag85A peptide-stimulated PBMC days 0 and 28	20	10
IFN-γ ELISpot (unstim, PHA, 85A) days 0 and 28	99	50
RNA-Sequencing: unstimulated PBMC days 0 and 28	12	6

GEX: gene expression analysis using Illumina Human HT-12 v4 microarray beadchips.

Day 0: day of vaccination with MVA85A/Candin placebo.

### MVA85A induces an inflammatory signature one day post-vaccination

**Figure 1:**
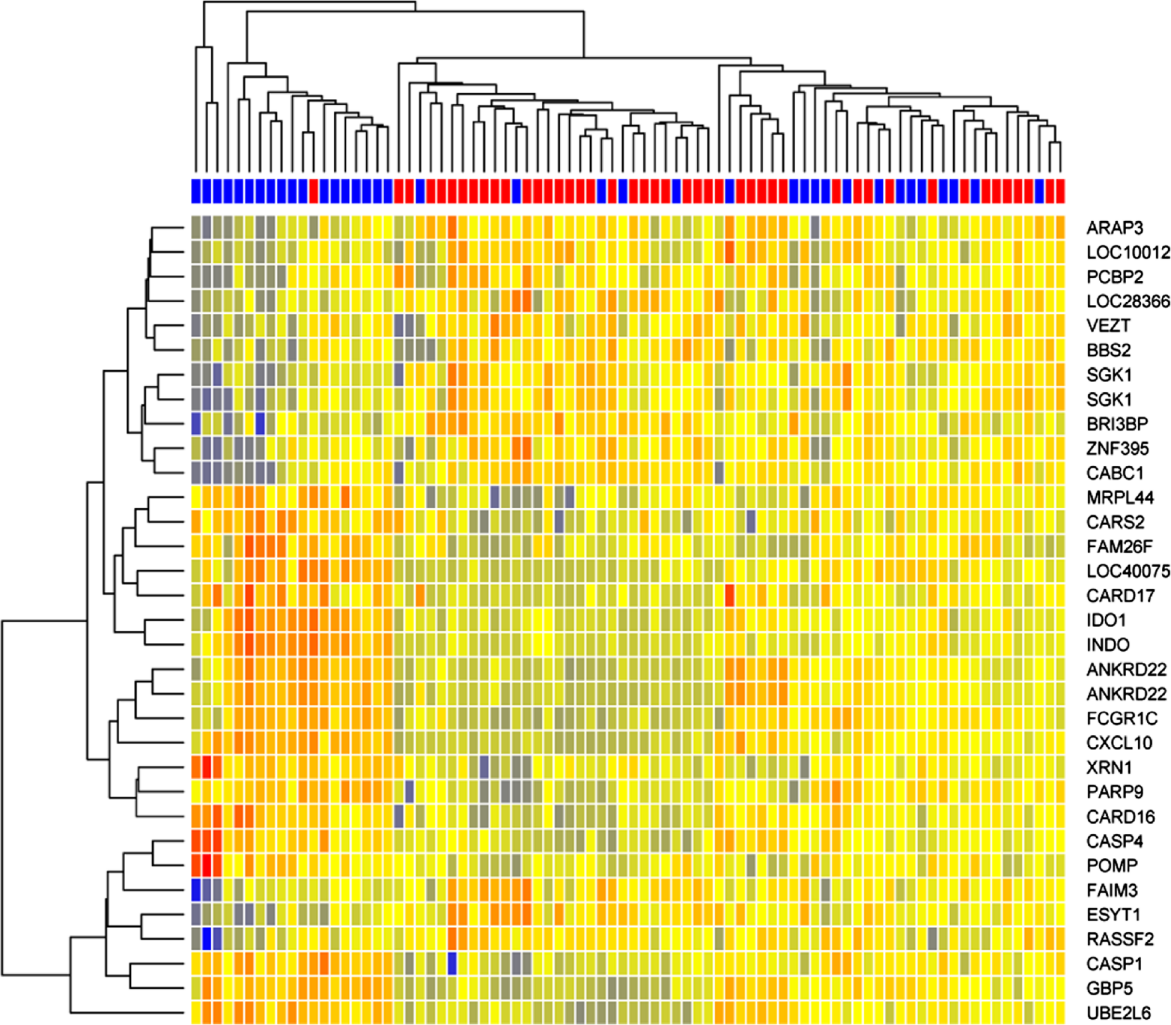
**Heatmap of differential gene expression 1 day post vaccination.** Heatmap showing changes in gene expression one day post-vaccination with either MVA85A or a Candin placebo. Colors at the top show vaccination: MVA85A (blue) or Candin (red). Genes were selected on the basis of differential expression between the two groups (fdr < 0.05). Clustering using euclidean distance and average clustering methods. Red indicates up-regulated mRNA, blue indicates downregulated.

Illumina microarray gene expression analysis of 82 whole blood samples taken one day post-vaccination (37 MVA85A, 45 Candin placebo) identified 32 differentially expressed genes. These genes were largely associated with the immune response (differentially expressed genes are shown in Table 2 with genes associated with the immune response highlighted in bold). Hierarchical clustering showed that infants fall into three clusters with a mixed MVA85A/Candin cluster exhibiting a moderate level of expression of inflammatory genes (Figure 1). This analysis shows MVA85A-vaccinated infants exhibit a more inflammatory gene expression profile than those in the placebo group however the range is large in both groups and there is an overlap between the two groups. This overlap could be due to the immunomodulatory properties of Candin, which induces inflammation and may lead to functional reprogramming of monocytes associated with protection from subsequent infection [34].

**Table 2:** Differentially expressed genes 1 day post-vaccination: MVA85A vs Candin

**PROBE_ID**	**SYMBOL**	**Fold change**	**AveExpr**	**adj.P.Val**
ILMN_1791759	**CXCL10**	3.2	7.18	0.04
ILMN_1799848	ANKRD22	2.61	6.65	0.04
ILMN_1656310	**INDO**	2.53	5.68	0.04
ILMN_2114568	**GBP5**	2.52	8.7	0.02
ILMN_2132599	ANKRD22	2.46	7.09	0.04
ILMN_3239965	**IDO1**	2.35	6.17	0.04
ILMN_3247506	**FCGR1C**	2.13	6.59	0.04
ILMN_1782487	LOC400759	1.96	5.38	0.01
ILMN_2066849	FAM26F	1.89	5.99	0.04
ILMN_1809086	XRN1	1.74	6.83	0.04
ILMN_2053527	**PARP9**	1.67	6.81	0.04
ILMN_1769520	UBE2L6	1.61	11.07	0.04
ILMN_1707979	**CARD17**	1.57	5.07	0.04
ILMN_2326509	**CASP1**	1.57	8.86	0.04
ILMN_1671452	MRPL44	1.5	6.05	0.04
ILMN_1700671	ETV7	1.49	4.83	0.04
ILMN_1678454	**CASP4**	1.48	9.83	0.04
ILMN_1715401	MT1G	1.43	4.78	0.01
ILMN_3238525	**CARD17**	1.35	4.6	0.04
ILMN_1693287	**POMP**	1.32	9.68	0.04
ILMN_1761159	ESYT1	-1.34	8.78	0.05
ILMN_1693410	BRI3BP	-1.37	6.56	0.04
ILMN_1774828	VEZT	-1.4	5.72	0.04
ILMN_3236036	LOC283663	-1.41	5.39	0.04
ILMN_3256478	LOC100129034	-1.41	5.38	0.04
ILMN_3240997	ARAP3	-1.43	5.7	0.04
ILMN_1767612	BBS2	-1.44	5.6	0.04
ILMN_1775542	**FAIM3**	-1.45	9.66	0.04
ILMN_3251155	PCBP2	-1.48	5.49	0.01
ILMN_1772876	ZNF395	-1.5	6.21	0.04
ILMN_3305938	SGK1	-1.56	6.59	0.04
ILMN_1731064	CABC1	-1.59	6.1	0.02
ILMN_3229324	SGK1	-1.6	6.3	0.04

Unstimulated whole blood, genes identified using the R package limma.

### The immune response to stimulation with Ag85A peptides

**Figure 2:**
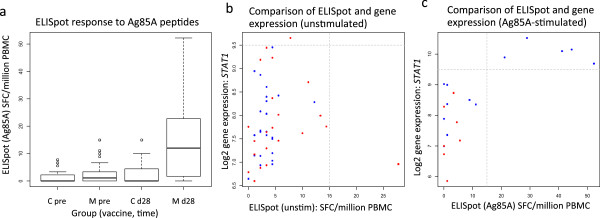
**Stimulation of PBMC with Ag85A peptide pools. (a)** IFN-γ ELISpot of PBMC taken pre- or post-vaccination in MVA85A or candin-vaccinated infants. Responses are significantly higher in the MVA85A group, 4 weeks post-vaccination (Wilcoxon test, p < 0.05). b,c. STAT1 expression in unstimulated **(b)** and 85A peptide-stimulated **(c)** PBMC. Squares = pre-vaccination, circles = 28 days post-vaccination; red = candin, blue = MVA85A. Dashed lines indicate a positive ELISpot response (x = 15) and elevated expression of STAT1 (y = 9.5).

The antigen-specific immune response to Ag85A was assessed by IFN-γ ELISpot and Illumina microarray gene expression analysis. Infants vaccinated with MVA85A had a significantly higher post-vaccination Ag85A-specific ELISpot response than the Candin group (Figure 2a). Differentially expressed genes between the vaccine and placebo groups, and pre- and post-vaccination time points, are shown in Table 3. The genes induced following Ag85A peptide stimulation are all associated with the STAT1 pathway and exhibit a highly correlated pattern of expression. Furthermore, upregulation of this pathway occurred in infants who also had a detectable Ag85A-specific T cell response by IFN-γ ELISpot assay but not in unstimulated cells or infants who received the candin placebo (Figure 2b,c). This observation suggests that, in this population, gene expression analysis does not add substantial information to that measured by the IFN-γ ELISpot assay in capturing the response to Ag85A peptide stimulation following MVA85A vaccination.

**Table 3:** Differentially expressed genes following Ag85A stimulation post-MVA85A vaccination

**Vaccinated infants: day 28- day-7**				**Day 28 samples: vaccinated- placebo**			
**SYMBOL**	**logFC**	**Fold change**	**adj.P.Val**	**SYMBOL**	**logFC**	**Fold change**	**adj.P.Val**
LOC400759	2.04	4.11	0.0047	LOC400759	2.71	6.55	0.012
GBP5	2.16	4.46	0.0079	STAT1	2.69	6.46	0.012
LOC730249	1.93	3.82	0.0079	STAT1	2.31	4.96	0.012
WARS	1.37	2.58	0.0079	CXCL10	3.41	10.64	0.015
ANKRD22	1.67	3.19	0.018	CCL8	4.02	16.21	0.027
GBP4	1.4	2.63	0.018	GBP4	2.19	4.55	0.027
WARS	1.25	2.38	0.018	CXCL9	1.95	3.86	0.027
TAP1	0.79	1.73	0.0234	STAT1	1.78	3.44	0.027
AIM2	1.05	2.07	0.0251	WARS	1.69	3.23	0.027
PSME2	0.72	1.65	0.0251	IFI35	1.49	2.8	0.027
CXCL9	1.63	3.09	0.0259	WARS	1.73	3.31	0.03
GBP1	1.9	3.72	0.0349	GBP1	2.86	7.28	0.031
STAT1	1.15	2.22	0.0354	PARP9	1.56	2.94	0.031
STAT1	1.32	2.49	0.0476	FBXO6	1.84	3.57	0.037
*STAT1*	*0.92*	*1.89*	*0.0746*	*PARP9*	*1.46*	*2.75*	*0.051*
*GBP1*	*1.87*	*3.66*	*0.0911*	*PARP14*	*1.24*	*2.36*	*0.051*
*CD38*	*0.8*	*1.74*	*0.0925*	*PSME2*	*1.12*	*2.17*	*0.051*
*CXCL10*	*2.3*	*4.92*	*0.1295*	*GBP5*	*2.65*	*6.3*	*0.058*
*LAP3*	*1.13*	*2.19*	*0.1295*	*EPSTI1*	*1.51*	*2.85*	*0.058*
*CEACAM1*	*0.96*	*1.95*	*0.1412*	*GBP1*	*3.01*	*8.05*	*0.062*
*PARP14*	*0.71*	*1.63*	*0.1412*	*IFNG*	*2.17*	*4.51*	*0.062*
*IFNG*	*1.67*	*3.19*	*0.1599*	*P2RX7*	*1.77*	*3.42*	*0.062*
				*SAMD9L*	*1.37*	*2.58*	*0.062*
				*PTER*	*1.18*	*2.27*	*0.062*
				*UBE2L6*	*1.14*	*2.21*	*0.076*
				*IFIT3*	*2.35*	*5.09*	*0.08*
				*SNHG5*	*-1.17*	*-2.25*	*0.08*
				*LOC730249*	*2.32*	*5*	*0.082*
				*PLA2G4C*	*1.39*	*2.61*	*0.082*

PBMC stimulated for 12 hours with Ag85A peptide pools, genes identified using the R package limma. Genes in italics have an adjusted p value 0.05 < p < 0.2).

A proportion of infants did not respond to antigenic stimulation with Ag85A peptides following MVA85A vaccination. This lack of response was observed both by IFN-γ ELISpot and gene expression analysis. It has been suggested that low or absent responses to MVA85A may be one explanation for the lack of efficacy observed in the trial [12]. Further analysis of cases and controls is under way to address this question however, in this smaller study, we next investigated some of the mechanisms underlying the magnitude of the adaptive immune response which develops following MVA85A vaccination.

### Myeloid cells and inflammation are associated with a higher ELISpot response

**Figure 3:**
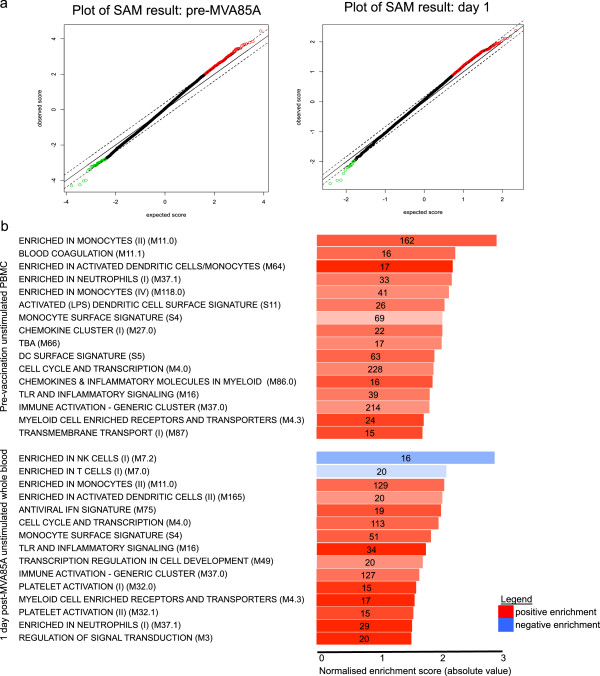
**Modular analysis of genes associated with a higher response to vaccination.** Genes whose expression correlate with the IFNg ELISpot response were identified using the R package SAM **(a)** and ranked in order of their score. The ranked list was then analysed in GSEA using the Blood Transcription Modules compiled by Li et al. as the reference gene set [33]. The results of the analysis for genes expressed in PBMC taken pre-vaccination or whole blood taken one day post-vaccination are shown **(b)**. Length of the bar shows Normalised enrichment score for each module, number in the bars indicates genes in the test list present ineach reference set. Colour saturation indicates genes present as a percentage of total genes within the module (signal). Red modules are positively associated with ELISpot response, blue modules are negatively associated.

**Figure 4:**
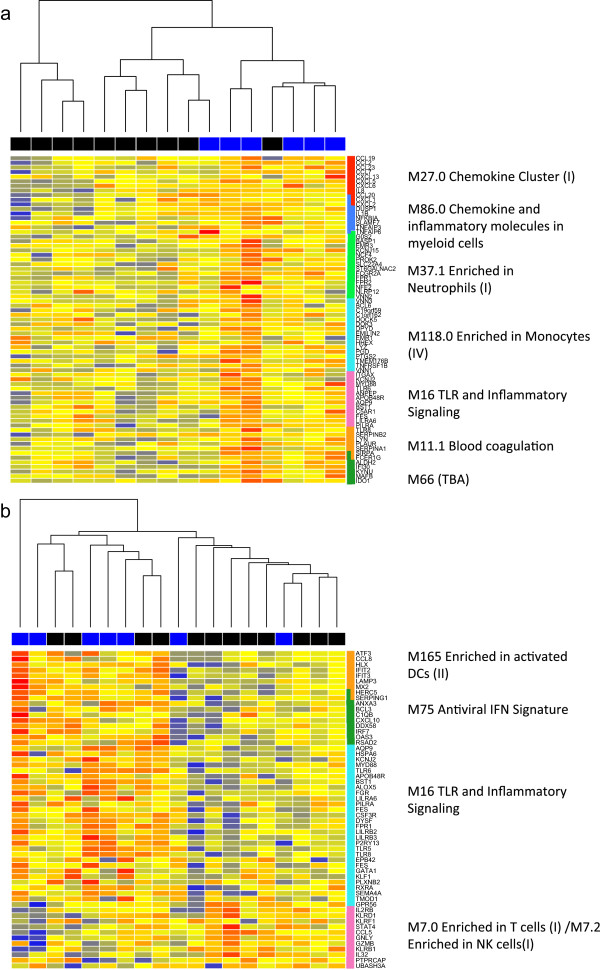
**Heatmaps of BTMs associated with a higher response to vaccination.** Heatmaps show expression of genes for MVA85A-vaccinated infants in unstimulated PBMC taken pre-vaccination **(a)** or whole blood taken one day post-vaccination **(b)**. The colour coding along the top of the heatmap shows responder (blue) and non-responder (black) infants as measured by IFN-γ ELISpot. Genes are arranged by module (right).

The following analyses were performed using only samples from infants vaccinated with MVA85A, to further investigate the mechanisms underlying the immune response to this vaccine. The R package Significance Analysis of Microarrays (SAMR) was used to identify genes whose expression correlated with the Ag85A-specific T cell frequencies measured 28 days post-vaccination by IFN-γ ELISpot assay. SAM identifies genes significantly correlating with a continuous response variable, in this case the IFN-γ ELISpot, and outputs a positive and negative set of genes based on the strength of the correlation of each gene with higher (positive) or lower (negative) values of the response phenotype [29,35]. Since the IFN-γ ELISpot responses in this study were very low, we have subsequently defined infants as responders or non-responders. An ELISpot response was deemed positive if the average count in the positive control wells was at least twice that in the negative control wells and at least 5 spots more than the negative control wells [36]. Sets of correlating genes pre-vaccination and one day post-vaccination were generated and ranked according to their significance score (Figure 3a). This ranked list of genes was then analysed using the Broad Institute Gene Set Enrichment Analysis PreRanked function, using the Blood Transcription Modules defined by Li et al. as a reference gene set [33]. Pre-vaccination, responder infants have an over-representation of genes enriched in monocytes, activated dendritic cells and neutrophils as well chemokines and inflammatory pathways (Figure 3b). One day post-vaccination, there is a negative association between lymphoid cells and the subsequent development of an ELISpot response (Figure 3c). Moreover, there is positive enrichment of gene sets associated with myeloid cells, inflammation and an antiviral response. The expression pattern of these clusters and their relationship to the ELISpot response is shown in heatmaps in Figure 4. Higher expression of genes associated myeloid cells and inflammation pre- and 1 day post-vaccination are both associated with the development of an antigen-specific T cell response to vaccination with MVA85A, suggesting the ability of MVA to induce a strong innate response is key to its function as a vaccine vector in this population. Additionally, higher expression of genes associated with activation of lymphoid cells such as NK cells and cytotoxic T cells one day post-vaccination is associated with an absent ELISpot response 28 days later.

**Figure 5:**
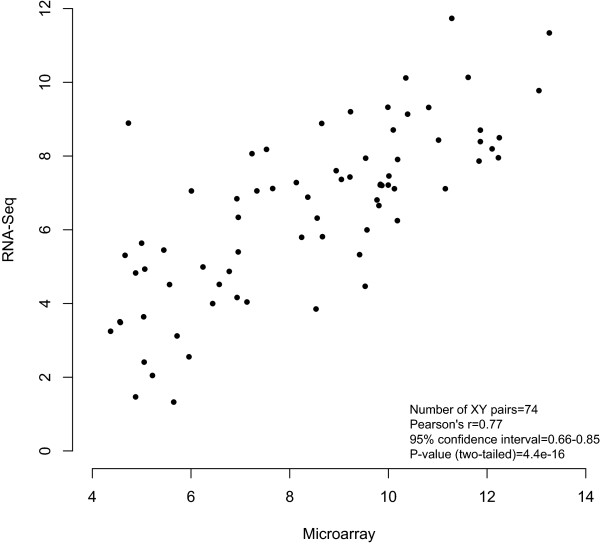
**Comparison of gene expression values measured by beadchip microarray and RNA sequencing.** Plot of median values from the RNA-Seq and microarray data of 12 unstimulated PBMC samples for 74 genes used in the modular analysis. Significance assessed using Pearson’s correlation.

The ratio of myeloid to lymphoid cells has previously been associated with differences in TB disease risk in HIV-infected South African adults and susceptibility to malaria and influenza in other cohorts [37-40] and this may be another example of an outcome associated with this ratio. The gene expression profiles associated with responder infants are present pre-vaccination and show a strong overlap pre- and one day post vaccination. This suggests the baseline inflammatory profile of the infant, including the differing proportion of circulating leukocytes, is key to determining the response to vaccination. This may be influenced by genetic influences on innate immunity or environmental exposure preceding vaccination, including the response to BCG, which all infants received at birth. As MVA preferentially infects myeloid cells [41], a higher proportion of myeloid cells may lead to overall increased viral expression of Ag85A protein in infants with higher frequencies of myeloid cells. Conversely, killing of infected myeloid cells by cytotoxic T cells and NK cells may decrease antigen expression, inhibiting the development of a response to Ag85A.In a subset of samples, the RNA extracted from the PBMC collected pre- and 28 days post-MVA85A was also measured by RNA Sequencing, as part of a pilot project for future studies. Gene expression values as measured independently by these two methods are highly correlated (Figure 5), providing a technical validation for this result.

### Gene expression signatures correlating with immunogenicity differ in South African infants and UK adults

We have previously described changes in gene expression in unstimulated PBMC from UK adults vaccinated with the same regime: a BCG prime followed by an MVA85A boost [23,42]. We therefore wanted to compare the observations made in these two different populations. In the adults, there was no placebo and PBMC were collected for gene expression analysis pre-, two and seven days post-MVA85A. Comparing gene expression to the magnitude of the induced T cell response showed a positive association with TLR1 expression at baseline and a negative correlation with regulatory genes including *STAT5B* and *CTLA4* at day 2 [42]. Table 4 shows the genes differentially expressed in infants one day post-MVA85A (compared to a placebo group at the equivalent timepoint). Genes in bold were also differentially expressed in UK adults two days post-MVA85A compared to baseline and fold changes are shown for both comparisons. In both populations, *CXCL10* had the highest fold change at the innate timepoint (one or two days post-MVA85A). One interesting difference is the gene encoding indoleamine 2,3-dioxygenase (probes *INDO* and *IDO1*) which is highly differentially expressed in the infants but not the adults.

**Table 4:** Comparison of differentially expressed genes in South African infants and UK adults post-MVA85A

	**Fold change**	
**SYMBOL**	**RSA infants**	**UK adults**
**CXCL10**	3.2	6.5
**ANKRD22**	2.6	2.0
INDO	2.5	1.3
**GBP5**	2.5	2.2
**ANKRD22**	2.5	1.7
IDO1	2.4	1.4
**FCGR1C**	2.1	1.7
**LOC400759**	2.0	2.1
**FAM26F**	1.9	1.8
XRN1	1.7	1.0
**PARP9**	1.7	1.8
**UBE2L6**	1.6	1.6
SGK1	-1.6	1.1
**CABC1**	-1.6	-1.3
CARD17	1.6	1.2
**CASP1**	1.6	1.7
SGK1	-1.6	1.1
MRPL44	1.5	1.1
**ZNF395**	-1.5	-1.3
ETV7	1.5	1.0
**CASP4**	1.5	1.2
PCBP2	-1.5	-1.2
**FAIM3**	-1.5	-1.3

South African infants: unstimulated whole blood taken 1 day post-vaccination. UK adults: unstimulated PBMC taken 2 days post-vaccination. All genes are differentially expressed between MVA85A and candin vaccinated infants (fdr < 0.05); genes in bold are also significantly different between day 0 and day 2 in UK adults vaccinated with MVA85A.

**Figure 6:**
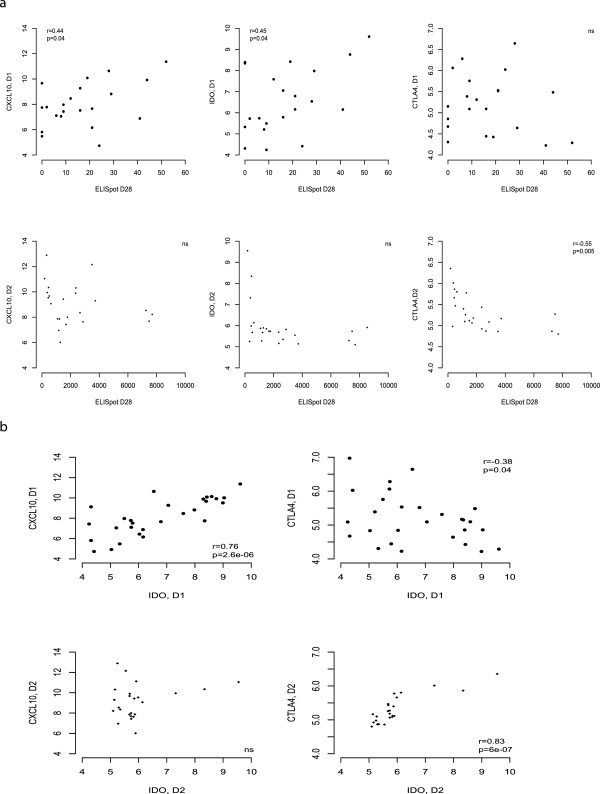
**Differences in inflammation and regulation: RSA infants and UK adults. a**. Expression in unstimulated whole blood 1 day post-MVA85A of CXCL10 and IDO1 but not CTLA4 correlates with ELISpot response in RSA infants. CTLA4 expression in unstimulated PBMC 2 days post-MVA85A is inversely correlated with ELISpot response in UK adults but IDO1 and CXCL10 show no significant association. **b**. In RSA infants, IDO1 expression correlates positively with CXCL10 and negatively with CTLA4 expression. In UK adults, CTLA4 and IDO1 expression show a positive association. All tests are Pearson’s correlations.

Further to this, the relationships between these genes show interesting differences between the two populations (Figure 6). In the South African infants, expression of both *CXCL10* and *IDO1* one day post-vaccination correlates with the IFN-γ ELISpot response and with each other. In the UK adults however, expression of these genes correlates neither with each other nor with the IFN-γ ELISpot response. In the UK adults, we have previously described a negative association between expression of *CTLA4* post-vaccination and the IFN-γ ELISpot response. However, this association was not found in South African infants. Furthermore, *CTLA4* correlated positively with *IDO1* in the UK adults but negatively in the South African infants.

Indoleamine 2,3-dioxygenase (IDO1) is an enzyme catalyzing the first and rate-limiting step in tryptophan catabolism. This enzyme has multiple physiological effects, including immunosuppression and regulation of T cells. In UK adults, expression of *IDO1* in PBMC correlates with that of the inhibitory co-receptor *CTLA4*. A recent study showing IDO1 is a critical resistance mechanism in antitumor T cell immunotherapy targeting CTLA-4 in a mouse model of melanoma suggests the interaction between these two genes is an important component to consider when inducing an immune response for therapeutic purposes [43]. In the South African infants however, we did not observe a correlation between expression of *IDO1* and *CTLA4* but *IDO1* correlated with *CXCL10* instead, suggesting either a different role for this enzyme or that inflammatory and regulatory responses are closely coupled in this population, perhaps to protect against excessive immune responses.

These disparities suggest there may be important differences in the immune response to vaccination in these two populations and in the pathways that determine the adaptive immune response. However, it is impossible to determine whether the differences observed are attributable to age or to genetic or environmental differences between the populations. Furthermore, the post-vaccination timepoint differed in both cell type (whole blood in the infants, PBMC in adults) and time taken (one or two days post-MVA85A). PBMC were used in the pre-vaccination timepoint in both studies. Despite this, the similarities in the list of differentially expressed genes suggest these comparisons are meaningful and warrant further investigation.

UK adults have a stronger innate response to the vaccine, however the magnitude of this response does not predict the magnitude of the antigen-specific response. By contrast, in the South African infants the magnitude of inflammatory gene expression one day post-MVA85A correlated with the ELISpot response at week 4. Further investigation of these mechanisms and how they differ between the different populations in which vaccines are tested and deployed may prove important in future vaccine development strategies*.*

## Conclusions

This study has shown an association between high levels of inflammation and myeloid signatures both pre- and post-vaccination and the development of the antigen-specific T cell response to MVA85A in BCG-vaccinated South African infants. In this population, the capacity of the vaccine to induce a strong innate response appears key to its ability to initiate an adaptive immune response. Furthermore, we describe differences in the response to vaccination with MVA85A in UK adults and South African infants, suggesting different immune pathways may determine immune responses in these two very different cohorts. This study has focused on investigating the mechanisms underlying vaccine immunogenicity, not vaccine-induced protection. Ultimately, an understanding of both these aspects of vaccination and how they differ across individuals and populations is likely to be necessary in achieving protection in the diverse groups and areas of the world still plagued by TB.

## Competing interests

The authors declare that they have no competing interests.

## Author contributions

MM, SAH, IS, LS, RT and MO carried out the experiments; MT, HMcS, MH, TJS, WAH and HAF were involved in study design and sample collection; MM analysed the data and drafted the manuscript. All authors reviewed and approved the final manuscript.

## Pre-publication history

The pre-publication history for this paper can be accessed here:

http://www.biomedcentral.com/1471-2334/14/314/prepub
